# Lateral inhibition in visual cortex of migraine patients between attacks

**DOI:** 10.1186/1129-2377-14-20

**Published:** 2013-02-28

**Authors:** Gianluca Coppola, Vincenzo Parisi, Cherubino Di Lorenzo, Mariano Serrao, Delphine Magis, Jean Schoenen, Francesco Pierelli

**Affiliations:** 1 Departmen of Neurophysiology of Vision and Neuroophtalmology, G.B. Bietti Foundation IRCCS, Via Livenza 3-00198, Rome, Italy; 2 Don Carlo Gnocchi Onlus Foundation, Lombardy, Italy; 3 Department of Medico-Surgical Sciences and Biotechnologies, “Sapienza” University of Rome Polo Pontino, Latina, Italy; 4 Headache Research Unit. University Dept. of Neurology & GIGA-Neurosciences, Liège University, Liège, Belgium; 5 IRCCS-Neuromed, Pozzilli, (IS), Italy

**Keywords:** Habituation, Migraine, Short-range lateral inhibition, Thalamo-cortical dysrhythmia, Visual evoked potentials

## Abstract

**Background:**

The interictal deficit of habituation to repetitive visual stimuli in migraine patients could be due to deficient intracortical inhibition and/or to low cortical pre-activation levels. Which of these abnormalities contributes more to the habituation deficit cannot be determined with the common methods used to record transient visual responses.

We investigated lateral inhibition in the visual cortex during the migraine cycle and in healthy subjects by using differential temporal modulations of radial windmill-dartboard (WD) or partial-windmill (PW) visual patterns.

**Methods:**

Transient (TR-VEP) and steady-state visual-evoked potentials (SS-VEP) were recorded in 65 migraine patients (21 without and 22 with aura between attacks; 22 patients during an attack) and in 21 healthy volunteers (HV). Three stimulations were used in each subject: classic checkerboard pattern (contrast-reversion 3.1Hz), WD and PW (contrast-reversion ~4Hz). For each randomly presented stimulation protocol, 600 sweeps were acquired and off-line partitioned in 6 blocks of 100. Fourier analysis allowed data to extract in SS-VEP the fundamental (1H) and the second harmonic (2H) components that reflect respectively short-(WD) and long- range lateral inhibition (attenuation of 2H in WD compared to PW).

**Results:**

Compared to HV, migraineurs recorded interictally had significantly less habituation of the N1-P1 TR-VEP component over subsequent blocks and they tended to have a smaller 1^st^ block amplitude. 1H amplitude in the 1^st^ block of WD SS-VEP was significantly greater than in HV and habituated in successive blocks, contrasting with an amplitude increase in HV. Both the interictal TR-VEP and SS-VEP abnormalities normalized during an attack. There was no significant between group difference in the PW 2H amplitude and its attenuation. When data of HV and migraine patients were combined, the habituation slope of WD-VEP 1H was negatively correlated with that of TR-VEP N1-P1 and with number of days since the last migraine attack.

**Conclusion:**

These results are in favour of a migraine cycle-dependent imbalance between excitation and inhibition in the visual cortex. We hypothesize that an interictal hypoactivity of monaminergic pathways may cause a functional disconnection of the thalamus in migraine leading to an abnormal intracortical short-range lateral inhibition that could contribute to the habituation deficit observed during stimulus repetition.

## Background

Deficient habituation of pattern reversal visual evoked potentials (VEPs) during stimulus repetition is a frequent characteristic of migraine patients during the pain-free interval [1-3]. The neural mechanisms of this habituation deficit are not fully elucidated. There are at least two possible, not mutually exclusive, explanations both supported by experimental data: hypofunction of intracortical inhibitory circuits [4,5] or reduced preactivation of the visual cortex by thalamocortical loops, secondary to hypoactive aminergic projections from the brainstem [1,2]. That the cortical dysexcitability in migraine could be due to abnormal thalamic control, namely to so-called “thalamo-cortical dysrhythmia” is suggested by studies of high frequency oscillations in spontaneous EEG and visual or somatosensory evoked potentials [6-8]. This concept may reconcile the advocates of excessive excitation and deficient inhibition, since a deficient thalamo-cortical drive, i.e. a low level of cortical preactivation, results in a dysfunction of both inhibitory and excitatory cortical neurons [9]. Reduced inhibition and low cortical preactivation may thus not be mutually exclusive, since the latter can promote the former through a reduction of lateral inhibition. Which of excitatory or inhibitory mechanisms contribute more to the habituation deficit cannot be determined with the methods commonly used to evoke visual responses that reflect the summation of both excitatory and inhibitory postsynaptic potentials [10].

Temporal modulation of adjacent regions of radial windmill-dartboard (W-D) or partial-windmill (P-W) visual patterns, however, allow to distinguish the relative contribution from short- and long-range lateral inhibition between cortical neurons during steady-state VEPs [11-13]. These visual patterns have permitted to identify two distinct phenomena in humans: a high amplitude response generated at the fundamental frequency band, and the attenuation of the second harmonic response during W-D stimulation relative to the second harmonic response during P-W stimulation. The fundamental frequency is thought to be generated by highly localized neuronal processes that result from lateral interactions over short distances (short-range lateral inhibition), whereas attenuation of the second harmonic component is due to lateral interactions between neurons over greater distances (long-range lateral inhibition) [13].

Using steady state VEPs obtained with W-D and P-W patterns, lateral inhibition in visual processing was already found abnormal in diseases like epilepsy [14,15] and schizophrenia [16].

The aim of this study was to explore whether lateral inhibition in the visual cortex is involved in the normal habituation phenomenon in healthy subjects and in the habituation deficit found in migraine patients between attacks.

## Methods

### Subjects

We enrolled 65 consecutive migraine patients (51 women), mean age 30.5 years, attending our headache clinic. Forty-three (21 without [MO] and 22 with aura [MA]) underwent VEP recordings during the interictal period, i.e. they were attack-free at least three days before and after the recordings. Twenty-two were recorded within a time range of 12 hours before or after the beginning of an attack. In this phase of the migraine cycle, close to an attack, the habituation deficit disappears showing a pattern similar to healthy controls [17-19]. This group of migraine patients were considered as ictal (MI). No preventive anti-migraine drugs were allowed during the preceding 3 months. For comparison, 21 healthy volunteers (HV) of comparable age and gender distribution (16 women, mean age 28.1 years) were recruited among medical school students and healthcare professionals. They had to be devoid of any overt medical condition, personal or family history of migraine or epilepsy, and regular drug intake. To minimize variability due to hormonal influences on cortical excitability, female subjects were always recorded at mid-cycle.

Participants taking medications on a regular basis and subjects who failed to reach a best-corrected visual acuity of > 8/10 were excluded. None of the enrolled subjects had sleep deprivation or alcohol ingestion the day preceding the recordings. Caffeinated beverages were not allowed on the day of recordings. All participants received a complete description of the study and granted written informed consent. The project was approved by the ethical review board of the Faculty of Medicine, University of Rome, Italy.

### Visual evoked potential recordings

#### Stimulation paradigms

**Figure 1:**
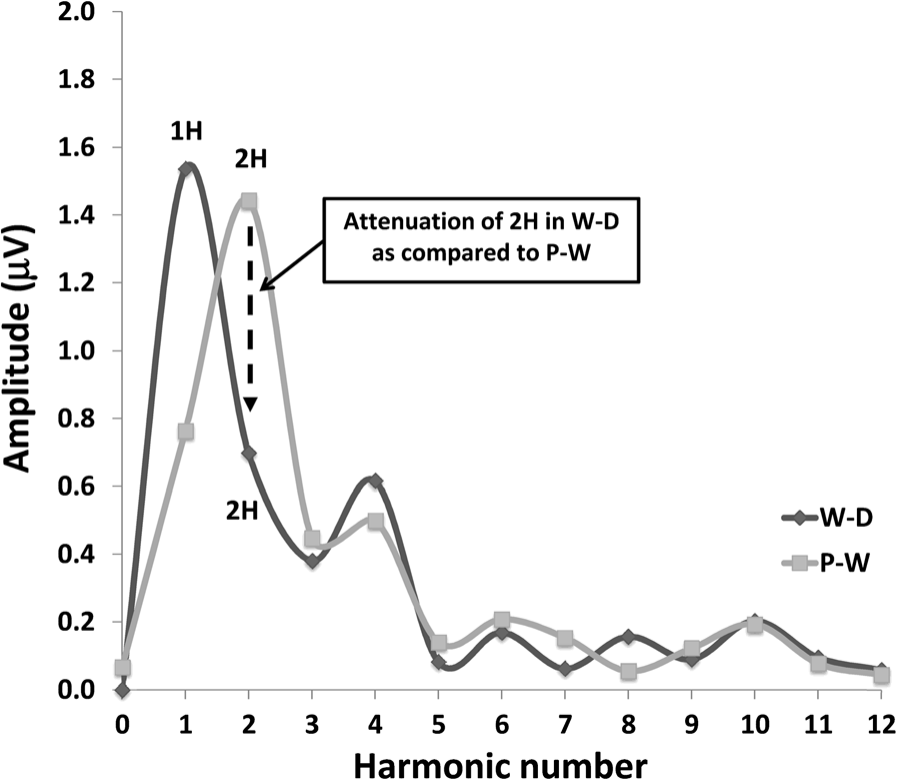
**Illustrative traces of visual evoked potentials obtained by checkerboard (left column)**, **windmill**-**dartboard (middle column) and partial**-**windmill (right column) stimulation in a healthy volunteer (upper row) and a migraine without aura patient between attacks (lower row).**

Subjects were seated in an acoustically isolated room with dimmed light in front of a TV monitor surrounded by a uniform luminance field of 5 cd/m^2^. To obtain a stable pupillary diameter, each subject adapted to the ambient room light for 10 min before the VEP recording. VEPs were elicited by right monocular stimulation. Three different stimulation paradigms were used. One consisted of a full-field checkerboard pattern (contrast 80%, mean luminance 250 cd/m^2^) generated on a TV monitor and reversed in contrast at a rate of 3.1/s (transient-VEP [TR-VEP]). At the viewing distance of 114 cm, the single check edges subtended a visual angle of 15 minutes. The other paradigms consisted of windmill-dartboard (W-D) and partial-windmill (P-W) patterns that were generated by using Presentation® software (Version 12.0, http://www.neurobs.com). The pattern is a central disc surrounded by three contiguous annuli, radially divided into light and dark segments [16]. The dynamic regions (central disc and 2^nd^ annulus) of both stimuli were sinusoïdally reversed in contrast at a rate of 4 Hz (steady-state VEP [SS-VEP]). At the viewing distance of 114 cm the stimulus display subtended a visual angle of 8°. In the W-D pattern, the black and white segments of the 1^st^ and the 3^rd^ annuli had a fixed contrast of 45%, so that the overall stimulus pattern alternated between the windmill and dartboard forms (Figure 1 top). Conversely, in the P-W pattern, the segments of the 1^st^ and the 3^rd^ annuli were set at a 0% contrast, forming a uniform field at the mean luminance level (Figure 1 top).

#### Recordings

During all stimulations, subjects were instructed to fixate a red dot in the middle of the screen with the left eye covered by a patch to maintain stable fixation. VEPs were recorded from the scalp through gold-cup electrodes positioned at Oz (active electrode) and Fz (reference electrode, 10/20 system). A ground electrode was placed on the right forearm. The evoked potential signals were amplified with a Digitimer D360 amplifier (band-pass 0.05–2000 Hz, Gain 1000) and recorded with a CED™ power 1401 device (Cambridge Electronic Design Ltd, Cambridge, UK). For each of the three stimulation paradigms a total of 600 consecutive sweeps of 240 ms were collected and sampled at 512 Hz. Cortical responses were partitioned in 6 sequential blocks of 100, consisting of at least 95 artefact-free sweeps. Responses in each block were averaged off-line (“block averages”) using the Signal™ software package version 4.08 (CED Ltd).

All recordings were collected in the morning (between 09.00 and 12.00 a.m.) by the same investigators (V.P. & M.S.) and off-line analysed blinded for subjects’ diagnosis.

Checkerboard TR-VEP components were identified according to their latencies: N1 as the most negative peak between 60 and 90 ms, P1 as the most positive peak following N1 between 80 and 120 ms, and N2 as the most negative peak following P1 between 125 and 150 ms (Figure 1). We measured peak-to-peak amplitude of the N1-P1 component.

SS-VEP (W-D and P-W patterns, Figure 1) were analysed in the frequency domain. After a discrete Fourier transformation (Figure 1), we measured amplitude of the fundamental harmonic (1H, at 4 Hz) for the W-D stimulus condition and of the 2^nd^ harmonic (2H, double the stimulus frequency, i.e. 8Hz) for both W-D and P-W stimulus condition [16]. To assess long-range lateral interactions, we calculated attenuation of the second harmonic component in the W-D respective to the P-W pattern.

### Statistical analyses

We used the Statistical Package for the Social Sciences (SPSS) for Windows, version 19.0 for all analyses. For each stimulus condition, we constructed a repeated analysis of variance (ANOVA) taking as within-subject factor “block” and as between-subject factors “Groups” (HV, MO, MA, MI). A regression analysis was used to disclose linear trends in VEP amplitude across blocks (slope) in each stimulus condition and group. Fisher’s least significant difference (LSD) test was used for post hoc analysis. Pearson’s correlation test was used to search for correlations among the VEP amplitude slopes and clinical variables.

P values ≤ 0.05 were considered to indicate statistical significance.

## Results

**Figure 2:**
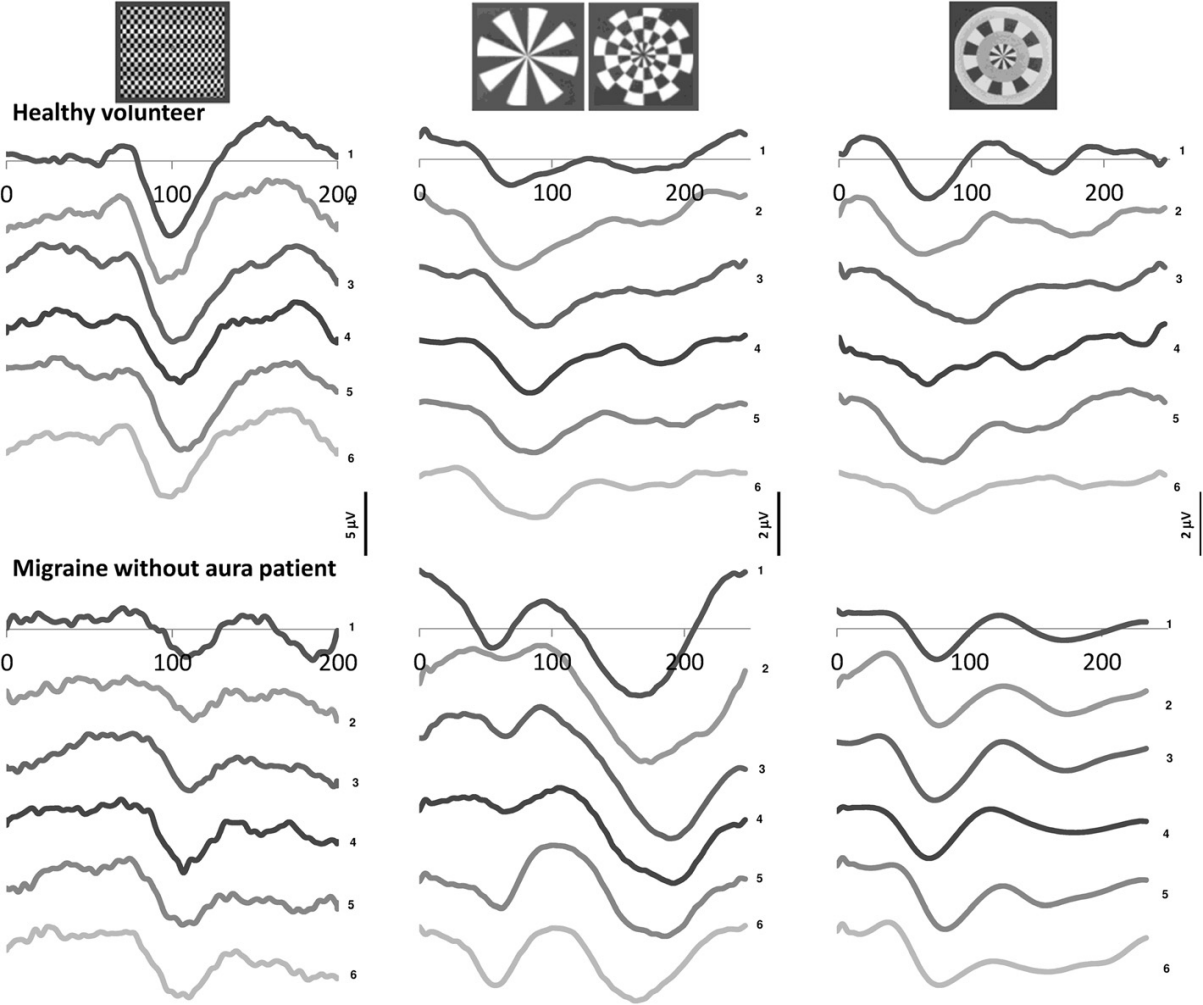
**Steady**-**state waveforms are converted into the frequency domain by Discrete Fourier Transformation in order to measure the amplitude at the first harmonic (4Hz, 1H) in the windmill**-**dartboard (W-D) stimulation**, **and of the second harmonic (8Hz, 2H) in both the windmill**-**dartboard and partial**-**windmill (W-D) stimulations.** To assess long-range lateral interactions, we calculated attenuation of the 2nd harmonic component in the W-D respective to the P-W pattern.

VEP recordings from all participants yielded analysable data. VEP traces recorded with the three different visual patterns in a healthy volunteer, a MO and a MA patient are illustrated in Figure 2. The clinical and demographic characteristics of recorded subjects are shown in Table 1.

**Table 1:** **Clinical and demographic characteristics of healthy volunteers** (**HV**) **and migraine patients without** (**MO**), **with aura** (**MA**), **and those recorded ictally** (**MI**)

***Characteristics***	**HV (n = 21)**	**MO (n = 21)**	**MA (n = 22)**	**MI (n = 22)**
Women (n)	16	17	19	15
Age (years)	28.1 ± 7.6	27.3 ± 6.5	30.8 ± 9.7	33.6 ± 11.9
Duration of migraine history (years)		13.8 ± 9.5	15.1 ± 8.8	19.7 ± 11.3
Attack frequency/month (n)		1.8 ± 1.1	2.1 ± 2.3	2.8 ± 2.5
Attack duration (hours)		27.3 ± 25.2	29.4 ± 23.7	33.1 ± 29.1
Days with headache/month (n)		3.3 ± 2.8	3.1 ± 3.2	4.2 ± 2.5
Days from the last migraine attack (n)		16.0 ± 23.4	17.0 ± 15.6	

Data are expressed as means ± SD.

### Transient-VEP (TR-VEP)

**Figure 3:**
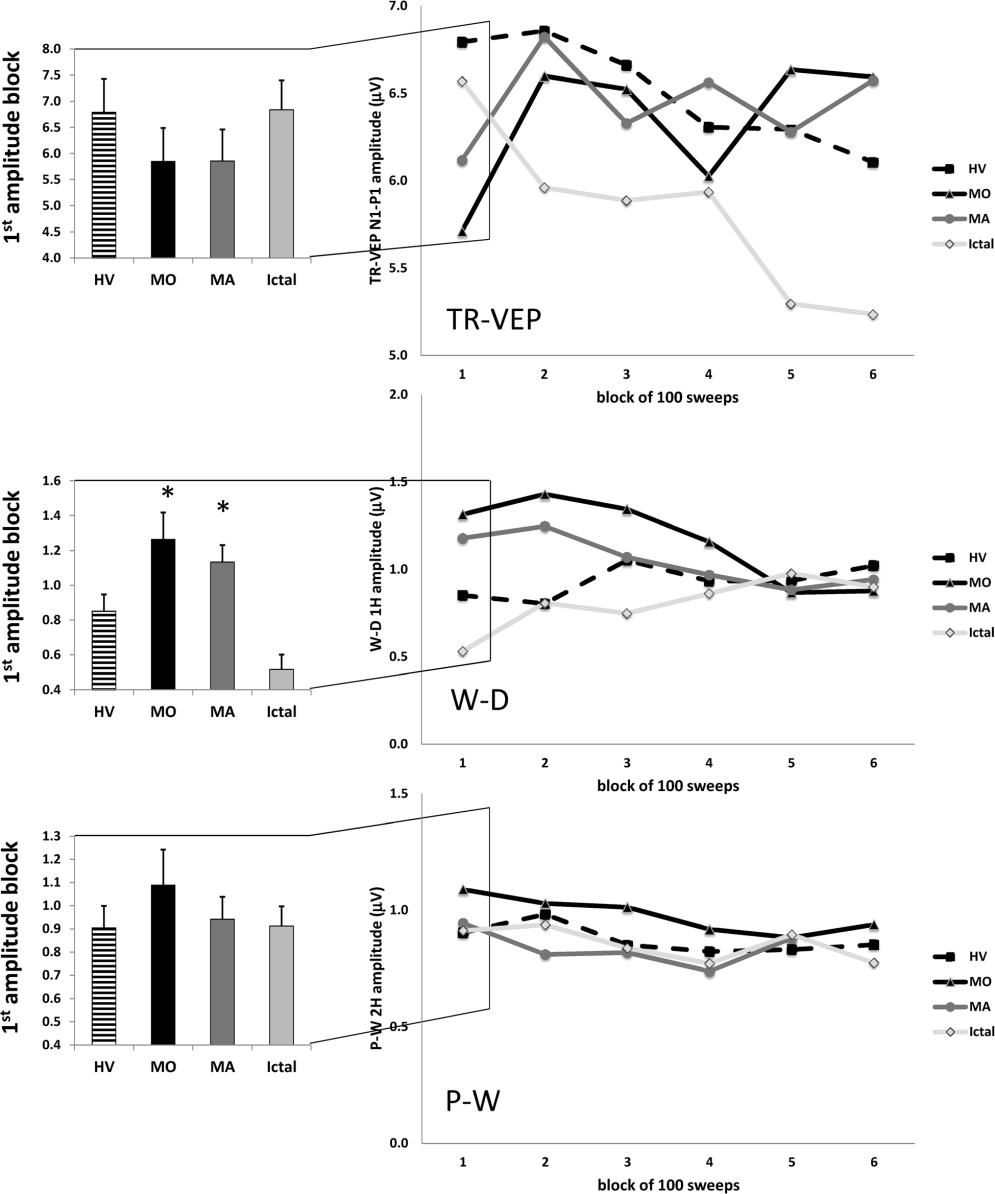
**Left panel: transient N1-P1 [upper], steady-state windmill-dartboard 1H [middle] and partial**-**windmill 2H [lower] mean amplitudes in the first block of 100 averaged responses; Right panel: Visual evoked potential (VEP) amplitude block averages in each study group and for the three types of visual stimuli: transient (TR-)VEP [upper], steady state windmill-dartboard (W-D) [middle] and partial-windmill (P-R) [lower] VEPs (HV, healthy volunteers; MO, migraine without aura interictally; MA, migraine with aura interictally; Ictal, migraine without aura ictally); data expressed as mean ± SD).**

**Figure 4:**
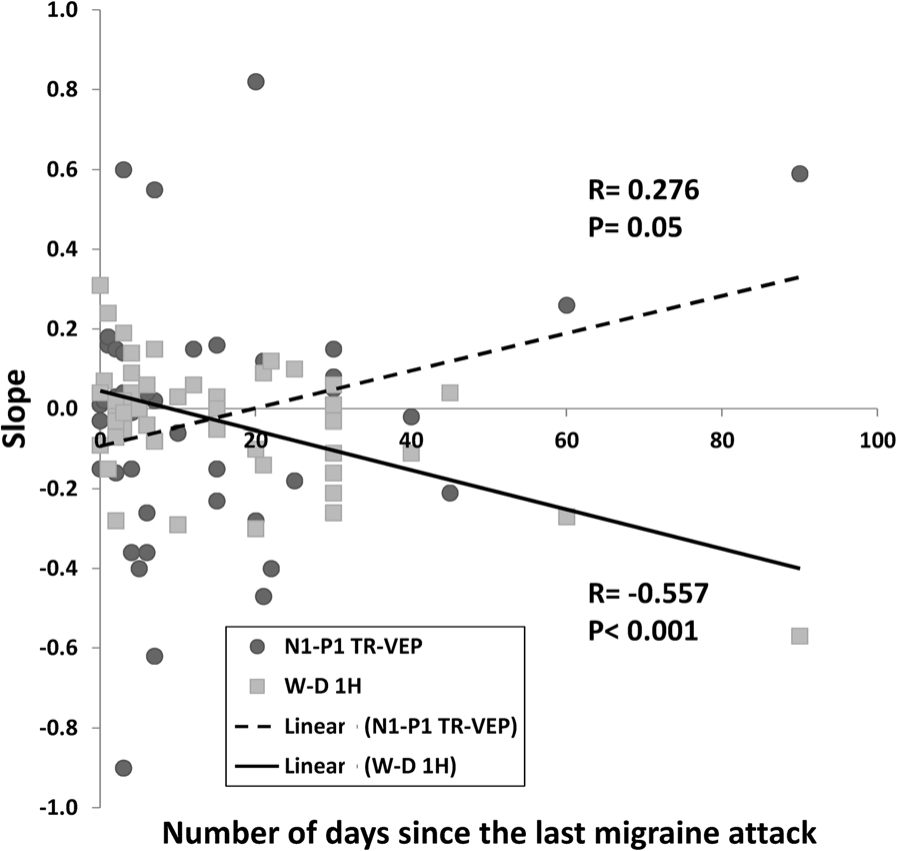
**Correlation between the number of days since the last migraine attack and the slope of 1H WD-VEP amplitude changes (negative, linear regression**: **uninterrupted line) or the slope of N1**-**P1 TR**-**VEP amplitude changes (positive, linear regression: dashed line) in the total cohort of migraine patients.**

ANOVA for N1-P1 amplitude in averaged TR-VEP blocks disclosed a main effect of factor block (F_(5,410)_ = 2.69, p=0.021) and a significant two-way interaction of group by block (F_(15,410)_ = 3.74, p<0.001) (Figure 3, upper row). N1-P1 amplitude in block 1 and 6 did not differed significantly between groups (F_(3,88)_ = 0.58, F_(3,88)_ = 1.43, p>0.05). Linear regression analysis of TR-VEP amplitudes recorded over all 6 blocks differed between groups (F_(3,80)_ = 9.66, p<0.001). Post hoc analysis showed that the slope of TR-VEP amplitudes from block 1 to block 6 was negative (−0.15 ± 0.20) in HV and in MI (−0.24 ± 0.23) whereas it was positive in patients recorded interictally (MO +0.11 ± 0.32, MA +0.02 ± 0.18; Figure 2 upper traces). When all patient groups (MO, MA, MI) were combined, there was a negative correlation on Pearson’s test between the amplitude slope of TR-VEP N1-P1 and duration of the migraine disease in years (r= −0.356, p=0.008) on the one hand, and monthly number of days with headache (r= −0.290, p=0.03) on the other. The amplitude slope was, however, positively correlated with the number of days since the last migraine attack (r= 0.275, p=0.05) (Figure 4).

### Windmill-Dartboard Steady-State-VEP (short-range lateral inhibition)

In the W-D condition ANOVA for 1H amplitude in averaged SS-VEP blocks disclosed a significant two-way interaction of group by block (F_(15,410)_ = 2.48, p=0.002), but not a main effect of group (F_(3,82)_ = 2.50, p=0.06) and block (F_(5,410)_ = 1.54, p= 0.17) separately. 1H amplitude in the 1^st^ block (F_(3,82)_ = 10.14, p<0.001) and slope of W-D VEP 1H amplitude changes over all 6 blocks (F_(3,82)_ = 10.75, p<0.001) differed between groups. Post hoc analysis showed that 1H amplitude in the 1^st^ block was significantly lower in HV (0.85 μV ± 0.43) and MI (0.53 μV ± 0.40) than in MO (1.31 μV ± 0.70, p=0.004 vs HV, p<0.001 vs MI) and MA (1.17 μV ± 0.45, p=0.04 vs HV, p<0.001 vs MI) (Figure 3). 1H amplitude in the 6^th^ block did not differed significantly between groups (F_(3,88)_ = 0.44, p>0.05). The slope of 1H amplitude variation from block 1 to block 6 was positive (+0.03 ± 0.10) in HV and in MI (+0.07 ± 0.08) but negative in patients recorded interictally (MO −0.11 ± 0.16, p<0.001 vs HV, p<0.001 vs MI; MA −0.06 ± 0.11, p=0.01 vs HV, p<0.001 vs MI; Figure 3, middle row).

**Figure 5:**
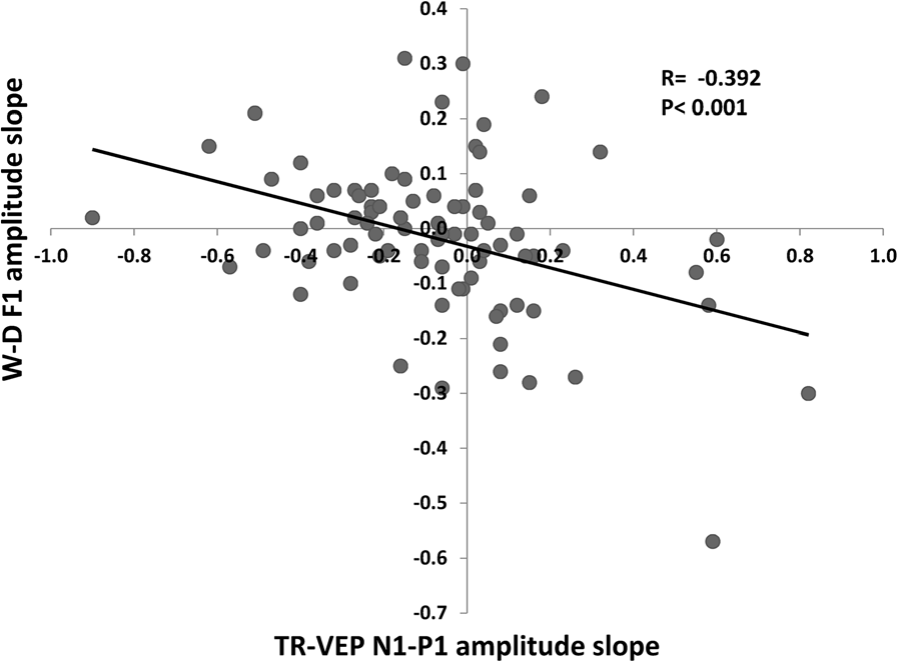
**Negative correlation between the habituation slope of 1H WD-VEP amplitudes and that of N1-P1 TR-VEP amplitudes in the total cohort of recorded subjects.**

In the entire cohort of patients (MO, MA, MI), Pearson’s test disclosed a negative correlation between amplitude slope of WD-VEP 1H and that of TR-VEP N1-P1 (r= −0.392, p<0.001) (Figure 4). The former was negatively (r= −0.557, p<0.001), the latter positively (r= +0.275, p= 0.05) correlated with the number of days elapsed from the last migraine attack at the time of recordings (Figure 5). Moreover, 1H amplitude in the 1^st^ block correlated positively with the slope of TR-VEP N1-P1 amplitudes (r=0.294, p=0.007), but not with amplitude in the 1^st^ TR-VEP N1-P1 block (r=0.028, p=0.803).

### Partial-Windmill Steady-State-VEP (SS-VEP)

In the P-W condition ANOVA for 2H amplitude in averaged SS-VEP blocks showed a significant main effect of factor block (F_(5,410)_=4.27, p=0.001), but not of group (F_(3,82)_=0.46, p=0.709) nor a two-way interaction of group by block (F_(15,410)_=0.57, p=0.897) (Figure 3, lower row). For the entire cohort of migraineurs combined there was no significant correlation between neurophysiological and clinical data.

### Long-range lateral inhibition

In order to assess long-range lateral inhibition we calculated the percentage change of the 2H amplitude between the P-W and the W-D conditions in each of the 6 sequential blocks of averagings. No significant main effect of group (F_(3,82)_ = 2.53, p=0.07) and block (F_(5,410)_ = 1.19, p=0.310) was detected, nor a two-way interaction of group by block (F_(15,410)_ = 1.07, p=0.377). There was no significant correlation between 1^st^ block amplitude or habituation slope and clinical variables.

## Discussion

We confirm in this study previous reports that TR-VEP 1^st^ block amplitude is decreased and does not habituate over subsequent blocks in migraineurs between attacks compared to healthy volunteers, but normalizes during an attack. The most striking result in our study, however, is that the degree of short-range lateral inhibition in the visual cortex of migraine patients differs from that of healthy volunteers. We found an increase in 1^st^ harmonic (1H) SS-VEP amplitude during windmill-dartboard (W-D) visual stimulation, reflecting the degree of short-range lateral inhibition, in migraine patients between attacks compared to healthy volunteers. Over successive blocks of recordings, however, short-range lateral inhibition decreases in migraineurs, while it increases in healthy controls.

During the ictal phase (MI), early short-range lateral inhibition, on the contrary, is much reduced compared to HVs, but it increases during stimulus repetition.

We found no difference between groups in long-range lateral interaction, as indexed by the attenuation of the 2^nd^ harmonic component (2H) between the W-D and P-W stimulation patterns.

We will discuss the possible neurobiological underpinnings of these data and their potential relevance for migraine pathophysiology.

Transient visual evoked responses recorded from the scalp are a complex mixture of electrical activities generated by multiple parallel functional channels within the visual system [20]. Nonetheless, by using adequate stimulation protocols it is possible to study specific VEP components. For instance, the relative contributions from lateral interactions between neurons of the visual cortex can be determined through differential temporal modulation of adjacent regions by radial windmill-dartboard (W-D) patterns [12,16,21]. The nonlinear cross-talk between the two neural areas that receive input signals at different frequencies of modulation (i.e., driven by the static or the dynamic stimulus zones) induces two distinct phenomena: a strong intermodulation response at the fundamental frequency (1H), and attenuation of the habitually dominant component at double the stimulus frequency (the 2^nd^ harmonic component, 2H). It is thought that the VEP component at the fundamental frequency elicited by the W-D stimulus is generated by a highly spatially localized phenomenon that is the result of lateral interactions occurring over small distances (“short-range lateral interactions”). By contrast, attenuation of the 2^nd^ harmonic component during W-D stimulation is the result of lateral interactions between neurons over longer distances (“long-range lateral interactions”) [13].

The lateral interactions in the visual cortex are attributable to inhibitory processes, possibly mediated by GABAergic synapses. According to the model first proposed by Hubel and Wiesel (1959), excitatory feed forward information coming from the periphery of the visual system travels through the thalamic lateral geniculate nucleus (LGN) and reaches simple and complex cells respectively in layers IV and III/II of the V1 area [22]. These cortical layers consist of spatial arrangements of direct-through excitatory inputs from the LGN, and lateral inhibitory pathways, orthogonal to the neurons’ excitatory input [22,23]. The visual cortex contains a dozen of inhibitory GABAergic cell types [24,25]. Amongst them, those with extremely restricted lateral axon bundles (<100 μm) are responsible for powerful local effects and thought to be the anatomical substrate of short-range lateral inhibition [26,27], while large basket cells possess the largest lateral axonal field (2 to 4 mm) and probably mediate long-range lateral inhibition [28].

Considering these anatomo-physiological data on the visual cortex, our findings suggest that in migraine there is a cycle-dependent abnormal dynamics of the excitatory-inhibitory balance in response to stimulus repetition.

Our finding of an interictal increase of the initial short-range lateral inhibition might explain some of our previous observations with TR-VEPs. Indeed, amplitude of the 1st block of TR-VEP is reduced in migraine patients between attacks but, like short-range lateral inhibition, normalizes during the attack [1,7,29-36]. An argument against this explanation, however, is that we found no correlation between the increase in 1st block 1H WD-VEP and the decrease in 1st block TR-VEP N1P1 amplitudes. Moreover, such an explanation might be too simplistic, considering that the TR-VEP reflects the balanced summation of both excitatory and inhibitory postsynaptic potentials [10]. We propose therefore that interictally in migraine there is an excessive prevalence of inhibition probably as a consequence of reduced excitation. The latter could account for the initial low amplitude of the TR-VEP N1-P1 component between attacks and its increase during attacks where the short-range lateral inhibition decreases and returns to normal, allowing excitatory connections to dominate. This interpretation receives support from various psychophysical studies exploring the balance between inhibition and excitation in migraine. With a pattern adaptation using visual illusions, a phenomenon attributed to a decrease in excitation from tonic pre-synaptic inputs [37,38], Shepherd et al. (2001, 2002) observed prolonged tilt after-effects, motion after-effects [39] and simultaneous tilt illusion [40] in MO and MA as compared to HV. This slow adaptation speed in migraine may indicate a lack of cortical excitatory connections [39-41], which consequently causes an increased strength of lateral inhibition in the visual cortex [42]. One cannot exclude the possibility that 1st block 1H WD-VEP is increased because of persistent hyperexcitability of pyramidal cells that in turn would also up regulate inhibitory circuits. In this case, one would expect significantly increased amplitude of the 1st TR-VEP block that would continue to increase in subsequent blocks, exceeding that of healthy volunteers. Our data, however, do not favor this hypothesis, since amplitude of the 1st TR-VEP block was normal or even tended to be lower in migraineurs, and its increase over the 6 blocks never exceeded the baseline amplitude of healthy volunteers (see Figure 3).

The second distinctive finding in our study is that interictally, after the initial increase, the 1H W-D decreased in amplitude, i.e. habituated over sequential blocks, contrasting with the amplitude increase, i.e. potentiation, found in HV. We propose that this habituation of short-range inhibition may contribute to induce the interictal lack of habituation of TR-VEP N1-P1 seen in migraine patients. This interpretation is supported by the inverse correlation we found between habituation of TR-VEP N1-P1 and that of the 1H SS-WD-VEPs: In other words, the more marked the VEP habituation deficit the more pronounced the habituation of short-range lateral inhibition. We must point out, that 1H amplitude in the last block did not differ significantly between groups (Figure 2). This indicates that it is the difference in the initial degree of short-range lateral inhibition that determines the ensuing increase of inhibition or excitation of pyramidal cells, which aim at restoring normal cortical activation. Such interpretation is in line with the concept of cortical homeostatic metaplasticity, postulating that whatever the initial level of cortical activation subsequent stimulations trigger a homeostatic mechanism by which the cortex tends to maintain its excitability level within a physiological range [43,44]. Taken together, our data suggest that the interictal deficient habituation in later VEP blocks in migraine strongly depends on the initial level of cortical activation. That the initial cortical performance is of importance in migraine is further evidenced by the results recently obtained in a visual-stimulus-onset asynchrony (SOA) psychophysical paradigm, the metacontrast masking test, attributed to inhibitory processes [45]. Shepherd et al. in an effort to reconcile the results from two previous contradictory studies [46,47], found that the apparently weaker metacontrast masking during visual SOA in migraine was due to the fact that both migraine groups performed differently in the baseline trials respective to controls. In fact, when baseline performance was taken into account, the group differences for each masking condition largely disappeared [45]. Our finding that the abnormalities of 1H and N1-P1 habituation increase with distance from the last migraine attack may be related to the ictal and peri-ictal changes described in thalamocortical connectivity [6] and cortical responsivity.

It is well known indeed that cortical responsivity fluctuates over time in relation to the migraine attack: just before and during the attack, habituation increases and normalizes [17]. TR-VEP N1-P1 habituation correlates inversely with the monthly number of headache days. This is probably due to the fact that patients with a high attack frequency are more likely to be recorded in a period close to an attack, i.e. at a time point when ictal changes have not completely disappeared [3]. Interestingly, a similar correlation with time elapsed since the last migraine attack was recently found with another measure of visual inhibition, the beforementioned metacontrast masking test [45]. This, together with our results, indicates that the overall inhibitory performance with stimulus repetition increases with proximity of the last migraine.

What causes the observed difference between impaired 1st harmonic responses to WD stimuli and normal 2nd harmonic responses to both PW and WD remains to be determined. Large basket cells, the mediators of long-range lateral inhibition, provide a dominant non-iso-orientation inhibitory input that could play a significant role in generating orientation tuning and direction selectivity [28]. With visual psychophysical paradigms using reaction times, evidence both in favour [48,49] and against [50] orientation-specific discrimination deficits were reported in migraine. Impaired orientation-specific discrimination, with normal detection threshold, was nevertheless present in several migraineurs when stimuli were presented at low spatial frequencies (less than 2 cycles/degree) [48,49]. We cannot thus rule out that the spatial frequency of 2 cycles/degree (15 min of arc) used in our study may have prevented us from detecting a significant impairment of long-range lateral inhibition in migraineurs which might have been possible with lower spatial frequencies.

**Figure 6:**
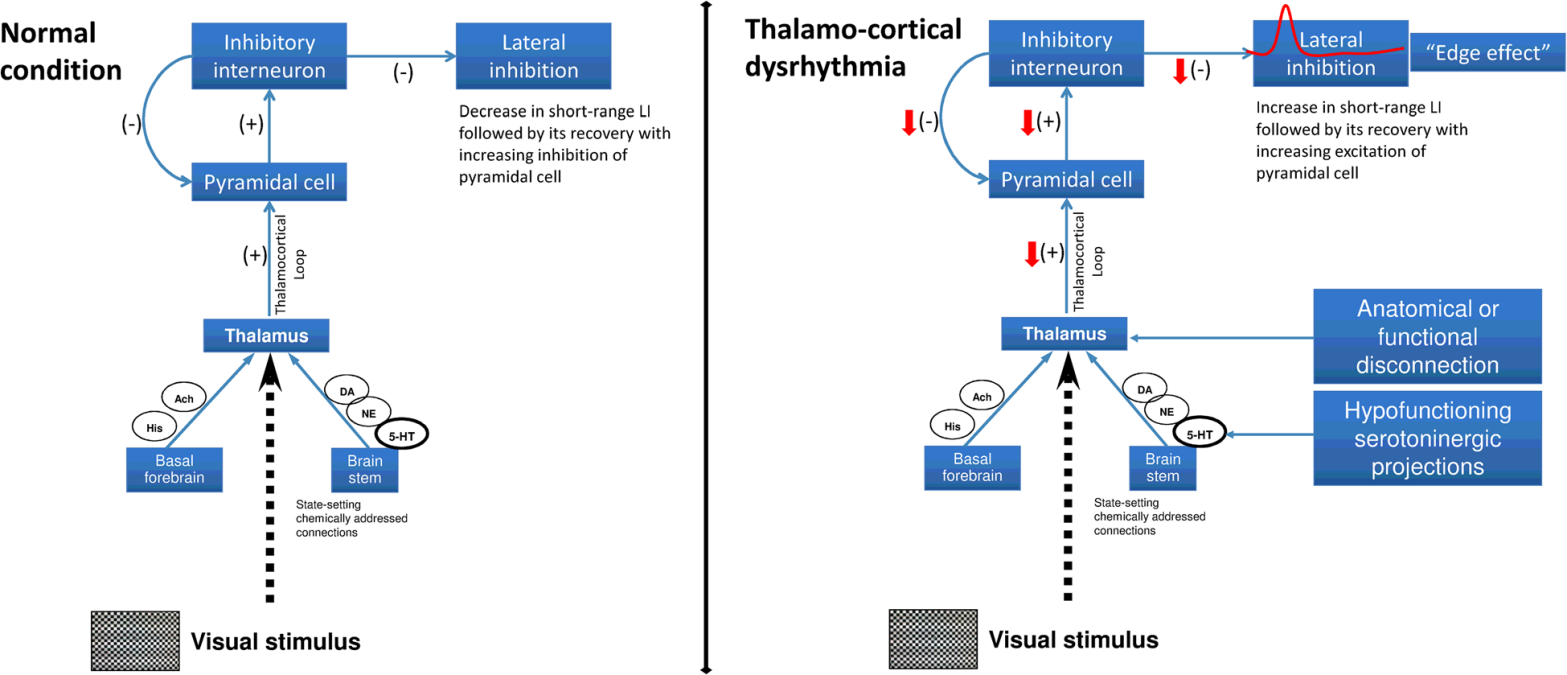
**Schematic representation of visual information processing pathway describing the neural network model that can encompass the present findings in both healthy volunteers and migraineurs.** In the normal condition (left panel), in presence of a regular brainstem and thalamic activation, visual information travels normally across subcortical areas, then at the cortical level increases the firing rate of excitatory pyramidal cells at the beginning and of fast-spiking (FS) inhibitory interneurons during stimulus repetition. The latter leads to a normal inhibition in adjacent cortical columns manifesting as a decrement in short-range lateral inhibition (LI) followed by its recovery with increasing inhibition of pyramidal cell (modified from [59]). In the thalamo-cortical dysrhythmia model (right panel), used to explain our findings in migraine, the presence of an anatomical or functional disconnection of the thalamus from subcortical areas, causes a change of rhythmic thalamocortical activity that favour low frequency activity which at the cortical level will reduce firing rates of excitatory pyramidal cells at the beginning and of FS inhibitory interneurons during stimulus repetition. The latter leads to a disinhibition in adjacent cortical columns manifesting as a progressive increase in short range LI, the so-called “edge effect”, which, in our migraineurs, is followed by its recovery with increasing excitation of pyramidal cells (modified from [60]).

A dysfunction of cortical inhibition has been described in a number of functional brain disorders grouped under the name “thalamo-cortical dysrhythmia” (TCD) syndromes. The TCD theory postulates that in presence of an anatomical or functional disconnection of the thalamus from subcortical areas, a change of rhythmic thalamocortical activity may occur favouring low frequency activity that at the cortical level will reduce firing rates of excitatory pyramidal cells at the beginning and of fast-spiking (FS) inhibitory interneurons during stimulus repetition [51-53]. The latter leads to a disinhibition in adjacent cortical columns manifesting as a progressive increase in high-frequency gamma band oscillations, the so-called “edge effect” [54,55]. Interestingly, FS inhibitory interneurons commonly make inhibitory electrical synapses with cells of the same type within a 100 μm inter-somatic distance (short-range lateral inhibition) [56]. Evidence favoring abnormal thalamic and thalamo-cortical activity in migraine between attacks also comes from studies of spontaneous EEG [8] and visual [7] or somatosensory evoked high-frequency oscillations [6,57]. We have proposed previously that hypofunctioning serotoninergic projections to thalamus and cortex might cause a functional disconnection of the thalamus leading to increased gamma band oscillations in the cortex, as in other thalamocortical dysrhythmias, and to reduced cortical habituation [2,7]. The role of serotonin transmission in migraine has been comprehensively reviewed by Hamel et al. (2007) and there is circumstantial evidence from clinical studies that migraine is a disorder with altered serotonergic transmission from the brainstem to the thalamus and cortex [58] (Figure 6).

## Conclusions

To conclude, we have shown that short-range lateral inhibition is impaired interictally in the visual cortex of migraine patients. We propose that this impairment may be responsible for the habituation deficit of transient VEP found in migraine and that it is likely a consequence of a functional thalamo-cortical disconnection due to a deficient serotoninergic innervation of thalamus and cortex. This may help to reconcile the controversy between abnormal excitation and deficient inhibition in interictal migraine. Indeed, some authors have proposed that the deficient habituation is due to a basal cortical lack of inhibition or increase of glutamatergic excitation [61-64]. At first glance, the fact that in our patients the amplitude of the short-range lateral inhibition was increased in the first block compared to healthy volunteers (see Figure 3) does not favour these hypotheses. Whereas, the observation that, after the early increase, the short-range inhibitory mechanisms abnormally decreased during long-term visual stimulation seems to fit better with the results provided by modulating rTMS studies, i.e. a reduced inhibition at long-term allowing excitatory connections to dominate [61-63]. Therefore, we propose that reduced inhibition and thalamo-cortical hypoactivity, i.e. low cortical preactivation, may coexist, since the latter can promote the former via reduction of lateral inhibition during the stimulus repetition. The final common pathway of both dysfunctions is a heightened cortical response to repeated stimuli, i.e. a lack of habituation.

Further studies are needed to confirm the present functional abnormalities and to determine their precise mechanisms, including its relation with changes in thalamo-cortical rhythms and in activity of subcortico-(thalamo-) cortical aminergic pathways. It will also be of uttermost importance to gather more data on the possible genetic basis of these functional abnormalities and their responsiveness to preventive anti-migraine interventions. The better insight into the nature of the interictal cortical hyperresponsivity paves the way for novel acute and preventive therapeutic strategies and may allow a better understanding of the mode of action of available pharmacological therapies and promising non-invasive neurostimulation methods such as repetitive transcranial magnetic or direct current stimulations [65].

## Abbreviations

EEG: Electroencephalography; FS: Fast-Spiking; H: Harmonic; HV: Healthy Volunteer; MA: Migraine with Aura; MI: Migraineur recorded during the attack (Ictal); MO: Migraine Without aura; PW: Partial-Windmill; RTMS: Repetitive Transcranial Magnetic Stimulation; SS: Steady-State; TCD: Thalamo-cortical dysrhythmia; TR: Transient; VEP: Visual Evoked Potential; WD: Windmill-Dartboard

## Competing interests

The authors declare that they have no competing interests.

## Authors’ contributions

GC made substantial contributions to interpretation of data as well as in drafting the manuscript. DM, VP, JS and FP were implied in the interpretation of data as well as in drafting the manuscript; CDL gave critical revision of the manuscript for important intellectual content. VP and MS were implied in recording and analyzing data. All authors read and approved the final manuscript.
